# Dramatic Regression of a Fungating Breast Lesion Treated with Radiation Therapy

**DOI:** 10.7759/cureus.1360

**Published:** 2017-06-16

**Authors:** Robert W Gao, Shane Edlund, Jianling Yuan

**Affiliations:** 1 School of Medicine, University of Minnesota; 2 Department of Radiation Oncology, University of Minnesota

**Keywords:** fungating breast lesions, palliative radiotherapy, locally advanced breast cancer

## Abstract

Although advances in screening mammography have dramatically improved the early detection of breast cancer, a subset of breast cancer cases still present as locally advanced disease. Some of these patients develop fungating lesions, which are difficult to manage and can have a severe impact on the quality of life. Palliative treatment options include surgery, intra-arterial chemotherapy, and radiation therapy. Herein, we describe the case of a patient who presented with a fungating breast mass and demonstrated an immediate and durable response to radiation therapy with a significantly improved quality of life.

## Introduction

Despite advances in breast cancer screening, locally advanced breast cancer (LABC) still represents 10-30% of new breast cancer diagnoses worldwide [1]. These cases may represent inherently aggressive disease or patient delay in seeking medical care. Among women with LABC, the subset of patients who develop a fungating mass in the breast is particularly challenging to manage. These malignant lesions have both ulcerative and fungating features and are often associated with pain, bleeding, malodorous drainage, and infection [2]. Regardless of the prognosis, these symptoms can have a devastating impact on patients’ quality of life and social wellbeing.

Initial management of a fungating breast mass often involves systemic therapy for cytoreduction, with the hope of eventual surgical resection and wound closure. However, when a lesion fails to respond or the patient becomes too debilitated to continue therapy, management options are very limited. Palliative measures, such as debridement, wound cleaning, dressing changes, and topical analgesics, may help reduce odor, pain, and infection [3]. Interventional techniques of angiography and endovascular embolization to control acute hemorrhage have also been described [4-5].

Radiation therapy is a commonly used modality in oncology and has proven efficacy in many palliative settings to relieve pain, stop bleeding, and reduce mass effect. Its effectiveness and safety in the management of fungating breast lesions, however, has rarely been reported. Here, we present the case of a 69-year-old female with a locally and systemically aggressive invasive ductal carcinoma who demonstrated an exceptional response to palliative radiotherapy to the breast.

## Case presentation

**Figure 1:**
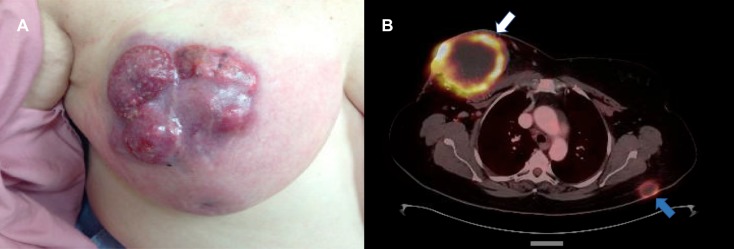
Initial Presentation of the Breast Mass (A) Physical examination revealed a 16 x 17 cm violaceous, nodular mass occupying the right breast with surrounding erythema. (B) Fluorodeoxyglucose positron emission tomography/computed tomography (FDG-PET/CT) scan showed a centrally necrotic and peripherally hypermetabolic enhancing soft tissue mass centered in the right breast with disruption and protrusion through the dermis (white arrow); also seen was a subcutaneous nodule (blue arrow).

A 69-year-old-female presented to her primary care provider in August 2014 with a one-month history of fatigue and bilateral lower extremity pain. Magnetic resonance imaging revealed abnormal marrow replacement in the bilateral proximal tibias. She was subsequently referred to our institution for an open biopsy. On the day of surgery, the patient revealed that she had developed a rapidly growing mass in her right breast over the past two months following a minor trauma. Physical examination showed a 16 x 17 cm violaceous, nodular mass occupying the right breast with surrounding erythema as well as palpable axillary adenopathy (Figure 1A). Pathology from the tibia biopsy demonstrated metastatic carcinoma felt to be compatible with a breast primary.

She subsequently underwent a core needle biopsy of the breast mass, which was consistent with invasive ductal carcinoma, Nottingham Grade 3, estrogen receptor 20% positive, progesterone receptor negative, and human epidermal growth factor receptor 2 amplified by fluorescence in situ hybridization. Fluorodeoxyglucose (FDG) positron emission tomography/computed tomography (PET/CT) demonstrated widespread disease, including lymph node involvement, subcutaneous nodules, and peritoneal masses throughout the chest, abdomen, and pelvis. Also seen were the known fungating right breast mass and bilateral tibial metastases (Figure 1B).

The patient began systemic therapy with weekly Taxol, trastuzumab, and pertuzumab to which she initially responded well with near resolution of the fungating portion of the mass. Her treatment course, however, was complicated by recurrent C. difficile colitis, bilateral pulmonary embolism, and Grade 3 peripheral neuropathy, necessitating the initiation of anticoagulation and discontinuation of Taxol. Shortly after, her breast mass re-grew significantly with markedly increased serosanguinous drainage. Her systemic therapy was switched to trastuzumab emtansine and pertuzumab. Due to a poor response, it was later changed to capecitabine and trastuzumab.

Around this time, the patient began to experience recurrent bleeding from the fungating mass. Conservative measures, including silver nitrate cautery and anti-fibrinolytic dressing, failed to control her bleeding. In February 2015, she underwent subselective particle embolization of the right lateral thoracic artery. In March, the procedure was repeated in the right internal mammary artery. An inferior vena cava filter was electively placed to reduce the anticoagulation requirement.

While embolization initially stopped the bleeding, she soon developed another bleeding site with continued aggressive growth of the fungating mass. Systemic therapy was once again changed to trastuzumab, everolimus, and vinorelbine. The patient refused further embolization due to severe post-procedural pain. She was not felt to be a candidate for palliative mastectomy and, therefore, was referred to us for consideration of palliative radiotherapy.

**Figure 2:**
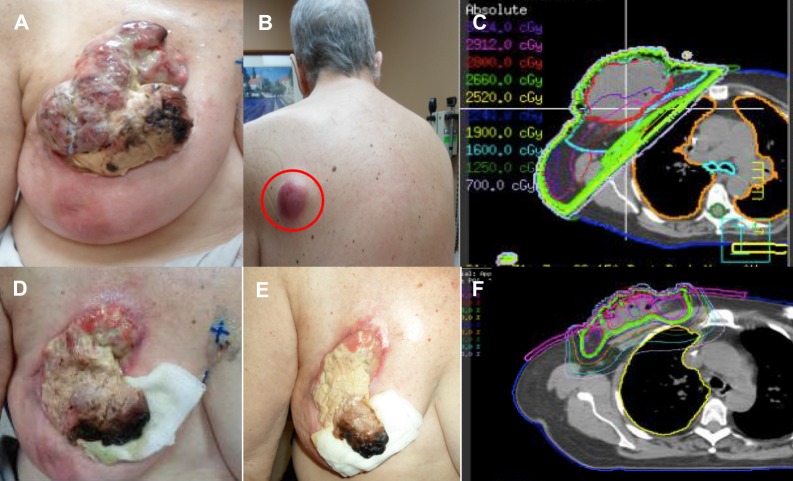
Radiation Therapy to the Breast Mass (A) Fungating mass at the time of radiation simulation; (B) An additional subcutaneous nodule in the left upper back (red circle); (C) A representative axial computed tomography slice showing the distribution of the radiation isodose line, 3,640 cGy over 13 fractions; (D) Appearance of the mass at the end of the first radiation course; (E) Mass at 10-day follow-up visit showing viable tumor in the superior aspect; (F) Electron beam therapy to the residual viable tumor, 1,500 cGy in five fractions.

On exam, the patient appeared withdrawn and teary. She had an impressive 15 x 10 cm fungating mass emanating from the right breast with necrotic tissue seen in the medial inferior aspect (Figure 2A). No foul smell or active bleeding was observed. She was also noted to have painful violaceous subcutaneous nodules over the left scapula, left flank, and forehead (Figure 2B). Radiotherapy was subsequently delivered to the right breast and symptomatic subcutaneous nodules. Her right breast mass initially received 3,640 cGy given over 13 daily fractions via 6 MV/10 MV tangential photons with a 0.5 cm bolus, while the skin nodules were treated with 2,000 cGy over five fractions using either photon or electron beam depending on the target depth (Figure 2C). Her right breast tumor regressed during treatment with significant portions of the exophytic mass becoming necrotic (Figure 2D). The patient returned 10 days later for a follow-up visit at which time further tumor regression was noted in both her breast and subcutaneous nodules (Figure 2E). However, there was clear evidence of viable tumor superiorly in her breast, and a decision was made to boost this area with an additional 1,500 cGy in five daily fractions using 15 MeV electron beams with a 0.5 cm bolus (Figure 2F).

**Figure 3:**
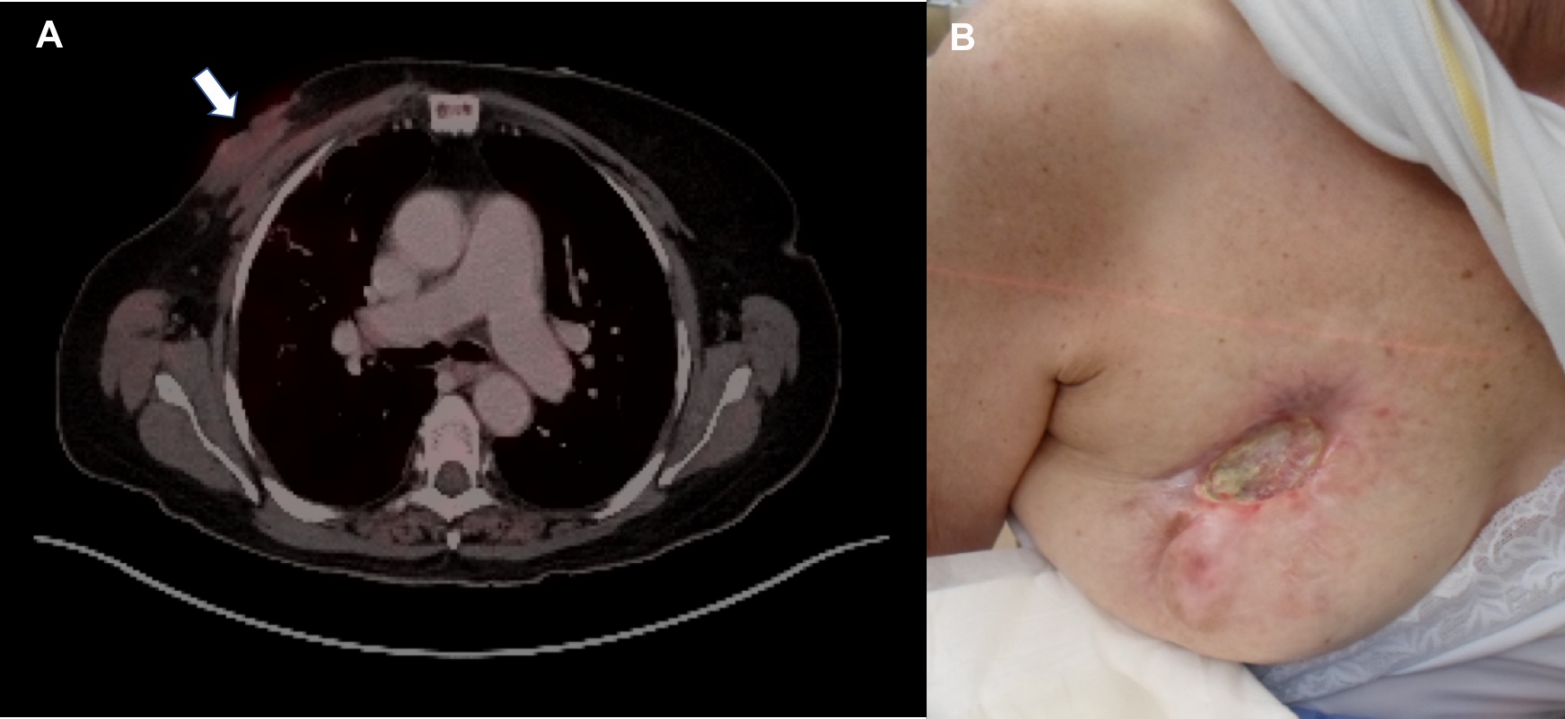
Response of the Breast Mass to Radiation Therapy (A) Fluorodeoxyglucose positron emission tomography/computed tomography (FDG-PET/CT) scan at four months after completion of radiotherapy (white arrow); (B) Regression of the mass and near closure of the open wound at four months post-treatment on physical exam.

She tolerated the radiation exceedingly well, experiencing only a mild skin reaction and no other side effects. Her excellent response to treatment was confirmed on physical examination and subsequent PET/CT scans (Figure 3A). At four months following treatment completion, her open wound nearly closed and no longer required time-consuming dressing changes (Figure 3B). She was able to resume her normal social life and was actively engaged in family events.

Unfortunately, the patient’s systemic disease continued to progress aggressively. Fifth line therapy with trastuzumab, lapatinib, and letrozole and sixth line therapy with trastuzumab and erilubin failed to control her disease. During this time, she received additional palliative radiotherapy for painful subcutaneous nodules and dural-based metastases with symptomatic improvement. Due to continued deterioration of her clinical status and lack of effective therapy, she elected to enroll in hospice care and ultimately succumbed to her disease in January 2016, 16 months following her initial diagnosis. At the time of her death, the breast mass remained controlled with no further bleeding, drainage, or pain.

## Discussion

Fungating breast lesions, although relatively uncommon, can present an enormous management challenge for health care providers. These malignant wounds are frequently associated with pain, mass effect, exudation, odor, pruritus, bleeding, crusting, and aesthetic distress [2]. Our patient experienced virtually all of these symptoms.

Many of these patients are not candidates for upfront surgical resection due to the extent of their disease, precluding effective wound closure. Systemic therapy may provide cytoreduction. However, not all patients can be successfully converted to surgical candidates. Although our patient initially responded to combination chemotherapy, Taxol had to be discontinued due to toxicity. None of the subsequent lines of systemic therapy provided notable disease control.

Her management was further complicated by the need for anticoagulation due to the development of a pulmonary embolism. This made control of her bleeding particularly difficult. While particle embolization likely provided some benefit, she continued to develop new bleeding from additional feeding vessels. The patient adamantly refused to undergo further intervention due to severe post-procedural pain.

As a last resort, the patient was referred for consideration of palliative radiation therapy. Her response to radiotherapy was quite dramatic. Bleeding immediately ceased and tumor regression was apparent during the treatment course. Although some of this response may have been due to antecedent embolization, the equally impressive response seen in similarly radiated subcutaneous nodules suggests a major therapeutic contribution from radiation. A positive palliative benefit from radiotherapy was also observed by Vempati, et al. who published a series of 13 patients receiving palliative treatment for ulcerative breast cancer [6]. Benefits of radiation included pain resolution, bleeding cessation, improvement of the wound, and the appearance of the site.

Due to the paucity of literature on this topic, the optimal radiation dose fractionation for effective treatment of fungating breast lesions is unknown. A dose in the range of 3,000 cGy over 10 fractions is commonly used in the palliative setting. For our patient, because of the bulk of the mass, we decided to increase the dose to 3,640 cGy in 13 fractions. While her tumor was significantly cytoreduced, there was still evidence of viable tumor, and this area was further boosted with 1,500 cGy in five fractions following a two-week break. Remarkably, the exophytic portion of the mass completely regressed and the wound was nearly closed. No significant toxicity was encountered despite the cumulative dose being approximately 55 Gy equivalent dose in 2-Gy fractions (EQD2). Consistent with our experience, Vempati, et al. observed a dose-response effect in their small series: six of the nine patients who received 30 Gy or more reported clinical benefit, whereas none of the four patients who received 30 Gy reported any benefit [6]. Although a definitive conclusion regarding dose fractionation cannot be generated from this small cohort, our experience along with the limited literature suggest that a minimum dose of 30 Gy, preferably upwards of 50 Gy, may be required to achieve an efficacious and durable response while minimizing toxicity. When selecting a dose regimen, one needs to be mindful of the incurable nature of the disease and the poor performance status of these patients. Minimizing recovery time and maximizing the quality of life is essential in this setting.

While our patient achieved excellent local control in the breast, her systemic disease progressed rapidly. At the time of her treatment, immunotherapy was not an available option. With continued understanding and development of immunotherapy strategies in recent years, targeted immunomodulators and immune checkpoint blockade have been increasingly combined with directed local radiotherapy to achieve an abscopal effect at distant non-irradiated sites [7]. The clinical efficacy of immunotherapy in conjunction with radiation therapy for breast cancer is not well established. However, several clinical trials are currently underway [8], the results of which may open the door for exciting new treatment options for women requiring management both at the primary and distant sites, such as our patient.

## Conclusions

In conclusion, the management of patients with fungating breast lesions remains a major therapeutic challenge. When systemic, surgical, and interventional radiological options are exhausted, radiation therapy can be an effective and safe option to achieve local control and reduce physical and psychological burden. Future research may be directed towards investigating the optimal dose and fractionation required to achieve a robust abscopal effect impacting not only local disease but also distant sites. 
